# Co-Occurrence of Systemic Lupus Erythematosus and Autoimmune Polyendocrine Syndrome II: Is There a Pathologic Link?

**DOI:** 10.7759/cureus.11187

**Published:** 2020-10-26

**Authors:** Sameen Aamer, Salman Akram, Muhammad Ali Butt, Aimal Shah

**Affiliations:** 1 Internal Medicine, Shifa International Hospital, Islamabad, PAK; 2 Internal Medicine, Rawalpindi Medical University, Rawalpindi, PAK; 3 Medicine, Nazareth Hospital - Trinity Health Mid-Atlantic, Philadelphia, USA

**Keywords:** autoimmune polyglandular syndrome, sle, systemic lupus erythematosus, pathology, autoimmune, endocrine disorders

## Abstract

Autoimmune polyendocrine syndrome type II (APS II) is a rare endocrine disorder that involves the adrenal gland (Addison’s disease), thyroid (autoimmune thyroiditis), pancreas (type 1 diabetes), and other non-endocrine organs. Herein, we report a case of a 58-year-old woman with a past medical history of systemic lupus erythematosus (SLE) and Addison’s disease, who initially presented with nocturia, polyuria, abnormal sweating, fatigue, hair thinning, heat and cold intolerance, and progressive darkening of the skin for the last few months. After a thorough evaluation, she was diagnosed with autoimmune thyroiditis, and thus, she met the criteria for APS II. This report highlights the unusual presentation of APS II in a patient with SLE. We also discuss common pathophysiological mechanisms that can explain the concurrence of SLE and APS II in this patient.

## Introduction

Autoimmune polyendocrine syndrome (APS) is a rare endocrine disorder [1], with the hallmark feature of lymphocytic infiltration of endocrine organs leading to a decrease in hormone production and symptoms of organ insufficiency. APS type II, a monogenic form, is characterized by Addison’s disease (occurring early in the course of the disease) along with thyroid autoimmune disease and/or type 1 diabetes. Like many autoimmune diseases, this syndrome is more prevalent in females, affecting females in their third and fourth decades of life [2]. Although APS II is primarily an endocrine disorder, it may also affect non-endocrine organs through autoimmune-mediated damage. Here, we report a case of APS type II, where the patient also met the criteria for systemic lupus erythematosus (SLE). We also discuss whether APS type II and SLE share common pathophysiological mechanisms.

## Case presentation

A 58-year-old woman with known cases of SLE, Addison’s disease, type 2 diabetes, and fibromyalgia presented to the clinic for the first time with concerns of polyuria, nocturia, abnormal sweating, excessive fatigue, hair thinning, heat and cold intolerance, and progressive darkening of the skin for the last few months. She was diagnosed with SLE at age 19 when she presented to her rheumatologist with concerns of polyarthralgia, hair loss, facial rash, rash on hands, weight loss, fatigue, headache, seizures, and photosensitivity, for which glucocorticoid therapy was initiated. The patient remained stable on glucocorticoid therapy, and there was no flare of the disease since then. At the age of 35, she was diagnosed with Addison’s disease and type 2 diabetes when she presented to the emergency department with hypoglycemia and an associated episode of loss of consciousness. A review of the patient's medication revealed that she was taking oral prednisone 2.5 mg, four times daily for Addison’s disease and SLE. A review of her family history showed that her aunt also had SLE. Physical examination showed vital signs within reference limits; her body mass index was 17.8 kg/m^2^, and she had epigastric tenderness and swollen gums. Based on the initial evaluation, laboratory tests were ordered consisting of anti-thyroglobulin antibodies, anti-microsomal antibodies, high-resolution computed tomography of the abdomen without contrast, and thyroid ultrasound. Moreover, hydrocortisone 10 mg twice per day was added to prednisone as part of the initial management.

**Figure 1:**
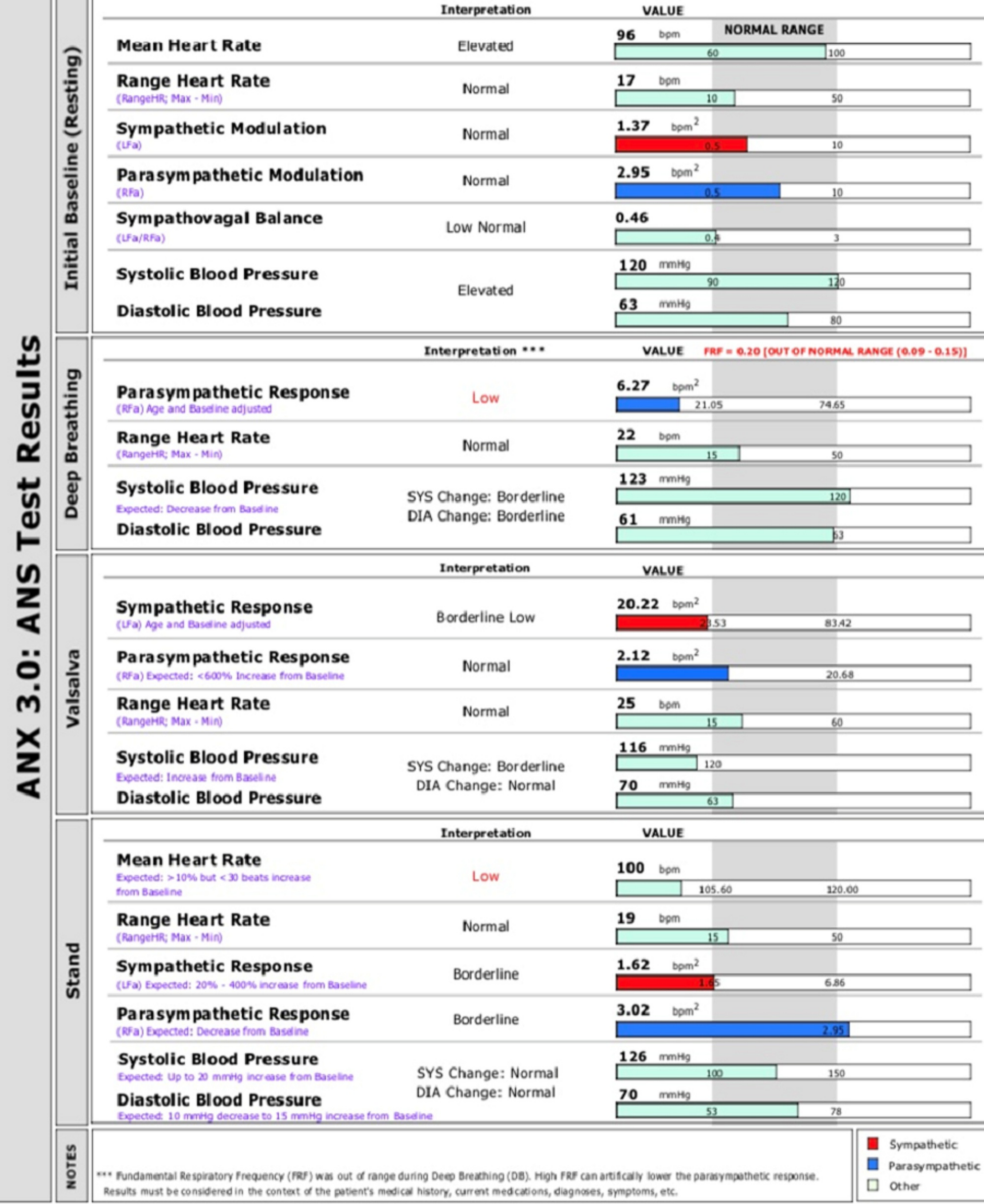
The patient's body response during Valsalva and deep breathing phase ANS, autonomic nervous system; SYS, systolic; DIA, diastolic; FRF, fundamental respiratory function.

Investigation results showed an increase in the size of the thyroid gland (an approximately 0.7-mm increase in the thickness of the left thyroid lobe) without any echogenicity or nodules and bilateral atrophic adrenal glands without discrete nodules. Thyroid-stimulating hormone (TSH) levels were in the high normal range (3.518 IU/L), serum antibody tests were positive for anti-thyroid peroxidase antibody and anti-thyroglobulin, and these tests, the patient’s symptoms, and other laboratory findings were all consistent with autoimmune thyroiditis. She was already diagnosed with Addison’s disease, and thus, she met the criteria for APS type II. Her oral, four times daily prednisone 2.5 mg tablet was discontinued, and she was advised to continue her oral, twice daily hydrocortisone 10 mg tablet. For the treatment of autoimmune thyroiditis, oral, once-daily levothyroxine was initiated (50 mcg tablet) in the morning, one hour before breakfast. The dosage of levothyroxine was later adjusted to optimize her TSH levels. The treatment regimen improved her symptoms and quality of life. Later, the patient complained of palpitations, tingling, and numbness. Therefore, to evaluate the patient’s concern, autonomic nervous system (ANS) testing was advised. The ANS test demonstrated abnormal responses to autonomic challenges (deep breathing, Valsalva, or standing), suggesting autonomic dysfunction (Figure 1).

Based on the results of ANS testing, the diagnosis for the patient was autonomic dysfunction. The patient was then evaluated every three months, and a yearly review of her laboratory test results is shown in Table 1.

**Table 1:** Patient's yearly laboratory results RBC, red blood cell; WBC, white blood cell; MCV, mean corpuscular volume; MCHC, mean corpuscular hemoglobin concentration; RDW, red cell distribution width; BUN, blood urea nitrogen; eGFR, estimated glomerular filtration rate; HDL, high-density lipoprotein; LDL, low-density lipoprotein; VLDL, very-low-density lipoprotein; TSH, thyroid-stimulating hormone; FT4, free thyroxine; T3, triiodothyronine; AST, aspartate transaminase; ALP, alkaline phosphatase; ALT, alanine aminotransferase; ACTH, adrenocorticotropic hormone; HBA1C, glycated hemoglobin.

Laboratory Test Parameters	2016	2017	2018	2019	Reference Range
Complete Blood Count					
RBC (X 10^6^)	4.58	4.52	4.47	4.59	3.77-5.28
WBC (X 10^3^)	8.7	12.2 (H)	11.3 (H)	8.5	3.4-10.8
Hemoglobin (g/dL)	12.7	14.0	13.3	13.3	11.1-15.9
Hematocrit (%)	39.0	40.7	41.7	40.3	34.0-46.6
MCV (fl)	89.0	90	93	88	79-97
MCH (pg)	29.0	29.4 pg	29.8	29.0	26.6-33.0
MCHC (g/dL)	32.6	32.7	31.9	33.0	31.5-35.7
Platelet count (x 10^3^)	194	239	249	285	150-379
RDW (%)	15.5	17.5	15.8	15.4	12.3-15.4
Total neutrophils (%)	71	73	66	59	Not established
Total lymphocytes (%)	18	17	25	6	Not established
Monocytes (%)	9	6	6	8	Not established
Eosinophils (%)	1	1	1	2	Not established
Basophils (%)	1	0	0	1	Not established
Neutrophils, absolute	6200	8800	7500	5000	1400-7000
Lymphocytes, absolute	1600	2100	2800	2500	700-3100
Monocytes, absolute	800	800	700	700	100-900
Eosinophils, absolute	100	100	100	200	0-400
Basophils, absolute	0.0	0.0	0.0	100	0-200
Basal Metabolic Profile					
Glucose, fasting (mg/dL)	93	101 (H)	86	99	65-99
Sodium (mmol/L)	145 (H)	142	146 (H)	138	134-144
Potassium (mmol/L)	4.0	4.4	4.6	4.1	3.5-5.2
Chloride (mmol/L)	106	96	102	99	96-106
Carbon dioxide (mmol/L)	26	24	26	21	20-29
Urea nitrogen (mg/dL)	23	13	22	16	6-24
Creatinine (mg/dL)	0.96	0.78	0.88	0.85	0.57-1.06
BUN/Creatinine	24 (H)	17	25 (H)	19	< 23
Calcium (mg/dL)	9.4	9.9	9.5	10.1	8.7-10.2
eGFR (ml/min/1.73)	> 60	> 60	> 60	> 60	> 60
Fasting Lipid Profile					
Cholesterol, total (mg/dL)	158	173	177	152	100-200
HDL cholesterol (mg/dL)	84	83	98	64	> 39
LDL cholesterol (mg/dL)	57	56	59	65	0-99
VLDL (mg/dL)	17	34	20	44 (H)	5-40
Triglycerides (mg/dL)	83	168 (H)	99	218 (H)	0-149
Thyroid Function Test					
TSH (µIU/L)	1.47	1.180	0.686	0.296	0.45-4.5
FT4 (ng/dL)	0.87	0.9	1.55	7.9	0.82-1.77
T3 (ng/dL)			98		
Hepatic Function Panel					
Protein total serum (g/dL)	5.2	6.4	6.1	6.5	6.0-8.5
Albumin (g/dL)	2.8	4.1	3.9	4.0	3.5-5.5
AST (IU/L)	19	15	13	35	0-32
ALP (IU/L)	69	59	82	108	50-117
ALT (IU/L)	22	14	18	34	0-40
Bilirubin, total (mg/dL)	1.02	<0.2	<0.2	0.3	0.00-0.45
Bilirubin, direct (mg/dL)	0.07	0.09	0.06	0.13	0.00-0.40
Microalbumin/Creatinine Ration, Random Urine					
Creatinine urine (mg/dL)		17.0		7.2	Not established
Microalbumin urine (µg/dL)		<3.0		<3.0	Not established
Microalbumin/Creatinine ratio (mg/g creatinine)		<17.6		ABNORMAL	0.0-30.0
Other					
Vitamin B12 (IU/L)	888	671	836	643	232-1245
Vitamin D25 (IU/L)	22.3 (L)	28.0 (L)	34.4	18.6 (L)	30.0-100.0
Cortisol, total serum (µg/dL)	39	26.1	58.0	13.1	
ACTH, plasma (pg/mL)	2.7 (L)	2.0 (L)	1.8 (L)	3.5 (L)	7.2-63.3
C-peptide, serum				3.8	
HBA1C (%)	6.3 (H)	6.0 (H)	5.9 (H)	6.0 (H)	4.8-5.5
Growth hormone (ng/mL)				0.9	0.0-1.0
Insulin, random (IU/L)		11.0			0.9-25

Due to her persistently decreased adrenocorticotropic hormone, her dose of oral hydrocortisone was decreased from two tablets to one tablet in the morning. Her type 2 diabetes was indicated by her C-peptide value and insulin levels in the reference ranges, and glycated hemoglobin slightly above the reference range (5.9% to 6.3%). The patient is subsequently being treated with dietary modification and exercise for type 2 diabetes. Other than this, throughout the course of the illness, the patient didn’t develop any signs or symptoms of other autoimmune manifestations of the disease; skin hypopigmentation (vitiligo), pernicious anemia (normal MCV and B12 levels), diarrhea (celiac disease), jaundice and abdominal pain (autoimmune hepatitis. Normal liver function tests), patchy hair loss (alopecia) and muscle weakness (myasthenia gravis), so further antibody workup for each of these systemic manifestations associated with APS type II was not done.

## Discussion

Our patient started visiting our endocrinology facility in 2013 after being referred by her primary care physician for the management of Addison’s disease and type 2 diabetes. Later, during her illness, she was diagnosed with autoimmune thyroiditis. Given this patient’s sex, age of disease onset, and the presence of Addison’s disease and autoimmune thyroiditis, she was diagnosed with APS II [2].

APS is a cluster of autoimmune disorders involving endocrine and non-endocrine organs, resulting in the manifestation of at least two autoimmune diseases based upon the lymphocytic infiltration of organ systems. Four subtypes of APS were described by Neufeld et al. in 1980 [3]. Two major subtypes, APS I and APS II, have been studied in much more detail than the other two types. APS I is characterized by chronic mucocutaneous candidiasis, hypoparathyroidism, and Addison’s disease. It usually manifests in infancy or early childhood, with a prevalence ratio of 1/100,000/year [4]. For this reason, it is also known as juvenile autoimmune polyendocrinopathy. APS II is more prevalent than APS I, with a prevalence ratio of 1-2/100,000/year [5], and usually occurs in adulthood. It is characterized by Addison’s disease, hypothyroidism, and/or type 1 diabetes. APS III is similar to APS II, except that Addison’s disease is absent in APS III. APS I and II also differ regarding their genetic basis, as APS II is a polygenic disorder, whereas APS I is a monogenic disorder (AIRE [autoimmune regulator] gene) [6].

The simultaneous occurrence of multiple endocrine disorders in APS II has been linked to the presence of specific haplotypes (e.g., class II human leukocyte antigen [HLA] haplotypes DR3 [DQB*0201] and DR4 [DQB1*0302]) [7]. The presence of DR3-DQ2 and DR4-DQ8 in patients with APS type II confer a risk of type 1 diabetes, autoimmune thyroid disease and Addison’s disease. In a study it was found that autoimmune Addison disease was strongly associated with HLA-DR3 and -DR4; relative risks were 6.0, 4.6, and 26.5 for DR3, DR4, and DR3/DR4, respectively [8]. Similarly, autoimmune thyroiditis and type 1 diabetes mellitus are associated with HLA DR 3-5, and HLA DR3 and DR4, respectively [9]. Recent studies also suggest that there is a complex interaction between non-HLA loci and environmental factors. APS II is inherited in an autosomal-dominant fashion with variable clinical expression. Other minor autoimmune diseases associated with APS II are vitiligo (5.7%), alopecia (3.8%), Sjogren’s syndrome (2.8%), pernicious anemia (1.9%), celiac disease (1.9%), autoimmune hepatitis (1.9%), and autoimmune thrombocytopenia (0.9%) [10]. These systemic manifestations also have an association with the HLA haplotypes. Celiac disease is associated with HLA DR3 [9]. Vitiligo, Sjogren Syndrome, Rheumatoid arthritis are also linked with HLA DR 4, and HLA DR3 and DR2, respectively [11,12]. This linkage explains why patients with APS type II may present with these autoimmune manifestations during the disease. APS IV consists of a combination of autoimmune disorders that are not included in the previous three APS types.

SLE is also autoimmune in origin and characterized by the formation of autoantibodies against the cellular nucleus and its components. Like APS type II, it also affects multiple organ systems, including the skin, joints, bone marrow, heart, lungs, kidneys, and brain [13]. According to the European Union against Rheumatism and the American College of Rheumatology [14], SLE is both a clinical and laboratory diagnosis, and the presence of a particular subset of clinical and laboratory parameters is required to diagnose a patient with SLE.

Although the exact genetic basis of systemic lupus erythematosus is still unknown, genome-wide association studies have determined that the strongest association of systemic lupus erythematosus is found within the human leukocyte antigen (HLA) region [15]. In SLE patients, different ethnicities are found to be increasingly susceptible based upon the presence of specific haplotypes [16], especially HLA-DR3 or DRB1*0301 in Europeans (and their descendants in North America and Australia), Tunisians, and Eastern Indians, HLA-DR3 in Latin Americans, and HLA-DR4 in Northern Indians.

Initially, the patient developed SLE, and then she developed Addison’s disease, autoimmune thyroiditis, and autonomic dysfunction. HLA-DR3 and HLA-DR 4 haplotypes are common to both SLE and APS type II. The haplotypes are not directly involved with the pathogenesis of the disease, but they are involved with the production of autoantibodies [16], and the presence of one autoimmune disease predisposes the patient to other autoimmune diseases. For example, the association of SLE with systemic sclerosis (SSc) was observed in one study that clearly showed that a haplotype of three different functional genetic variants within the Interferon Regulatory Factor 5 region conferred susceptibility to SSc. The fact that this association is shared with SLE adds another piece of evidence to the common genetic background of both diseases [17].

Similarly, a study performed on SLE patients in Taiwan showed the involvement of the thyroid gland [18], and a case report published in 2014 revealed that SLE can be associated with adrenal insufficiency [19]. Therefore, it is worth exploring the possibility that there are shared molecular pathways between the two disorders (SLE and APS II), and there is also a possibility that SLE predisposed the patient to APS II and autonomic dysfunction. A case report published in 2001 showed the simultaneous presence of SLE and APS II [20], but in our case report, in addition to the presence of both SLE and APS II, the patient also had autonomic dysfunction that had not been previously reported with SLE and APS II. Moreover, our case report documents the association between SLE and APS type II in an adult, on the basis of genetic linkages, which hasn’t been discussed before.

## Conclusions

APS II is a rare endocrine syndrome. SLE is not a rare rheumatologic disease, yet it is also autoimmune in nature. Autoimmune diseases affect multiple organs through the production of autoantibodies, and HLA haplotypes influence the formation of autoantibodies. Because both APS II and SLE are associated with HLA DR-3 and HLA DR-4, there is a possibility that the development of SLE early in life predisposed the patient towards the development of APS II later in life due to shared associated HLA haplotypes.
